# Sleep Duration and Sleep Quality Are Associated with Physical Activity in Elderly People Living in Nursing Homes

**DOI:** 10.3390/ijerph15112512

**Published:** 2018-11-09

**Authors:** Lovro Štefan, Goran Vrgoč, Tomislav Rupčić, Goran Sporiš, Damir Sekulić

**Affiliations:** 1 Faculty of Kinesiology, University of Zagreb, Zagreb 10000, Croatia; tomislav.rupcic@kif.hr (T.R.); sporis.79@gmail.com (G.S.); 2 Clinical Hospital Center ‘Sveti Duh’, Zagreb 10000, Croatia; gvrgoc@gmail.com; 3 Faculty of Kinesiology, University of Split, Split 21000, Croatia; damirsekulich@gmail.com

**Keywords:** sleep, exercise, older adults, associations, logistic regression, public health

## Abstract

The main purpose of the study was to explore the associations of sleep duration and sleep quality with physical activity (PA). In this cross-sectional study, participants were 894 elderly individuals (mean age 80 ± 3 years; 56.0% women) living in nursing homes. PA, sleep duration, and sleep quality (based on the Pittsburgh Sleep Quality Index (PSQI)) were self-reported. The associations of sleep duration and sleep quality with PA at the nursing home level were analyzed using generalized estimating equations with clustering. Participants reporting short sleep duration (<6 h; OR = 0.45; 95% CI 0.25–0.80) were less likely to report sufficient PA, yet those reporting long sleep duration (>9 h; OR = 2.61; 95% CI 1.35–5.02) and good sleep quality (<5 points; OR = 1.59; 95% CI 1.19–2.12) were more likely to report sufficient PA. When sleep duration and sleep quality were entered into the same model, the same associations remained. This study shows that elderly individuals who report short sleep duration are less likely to meet PA guidelines, while those who report long sleep duration and good sleep quality are more likely to meet PA guidelines. Strategies aiming to improve sleep duration and sleep quality are warranted.

## 1. Introduction

Physical activity (PA) represents an important factor for maintaining health in the elderly [1]. Elderly people are almost two times more likely to have physical or mental disabilities and are four times more likely to have some physical limitations, compared with people aged <60 [2]. Thus, encouraging regular PA in elderly people has been the goal of many governments worldwide [3,4] in order to prevent certain diseases and reduce mortality rates. The World Health Organization (WHO) guidelines propose that elderly people aged ≥65 should participate in “at least 150 min of moderate-intensity aerobic PA throughout the week or do at least 75 min of vigorous-intensity aerobic PA throughout the week or an equivalent combination of both” [5]. It has been-well documented that regular participation in PA leads to reduced incidence of cardiovascular, metabolic, and musculoskeletal disorders, and reduced overall mortality [6].

Sleep duration is among the numerous factors that can potentially influence PA [7]. Previous studies have reported that elderly people aged ≥65 suffer from a variety of sleep problems [8] including both short and long sleep duration. Specifically, Tsou [9] conducted a study among elderly Taiwanese aiming to explore the association between sleep duration and health-related outcomes. Among numerous factors, the author found that participants who were “long” sleepers (≥9 h) were less likely to exercise on a regular basis. Another study conducted among Chinese community-dwelling elderly people showed that both short and long sleep duration was associated with poor physical performance, compared to individuals reporting 7–8 h of sleep [10].

Along with sleep duration, sleep quality has also been associated with PA [11,12,13]. Findings of previous cross-sectional [11,12] and longitudinal studies [13] have shown that poor sleepers are less likely to meet PA guidelines [11,12] and that better initial sleep quality predicted higher levels of later PA [13]. In general, the association between sleep and PA is bidirectional [14].

In Croatia, studies about elderly populations are scarce. One previous study using the International Physical Activity Questionnaire–long form (IPAQ–long form) showed that individuals aged ≥65 were highly physically active in domestic and garden activities, yet were the least active in the leisure-time domain [15]. In general, studies have shown that the level of PA declines between age groups 65–69 and ≥85 for 10–25% of individuals, and point out that strategies and policies aiming to promote PA in the elderly are warranted [16]. On the other hand, evidence shows that sleep complaints and problems related to sleep increase with old age [17], with some studies reporting the prevalence of sleep complaints as 50% [18]. However, elderly people need optimal sleep duration and high sleep quality in order to perform both physical and psychological functions properly. Although numerous studies have shown the associations between physical activity and sleep [19,20], little is known about whether sleep predicts physical activity, especially in elderly populations.

Therefore, the main purpose of the study was to explore the associations of sleep duration and sleep quality with PA. The first hypothesis was that elderly people reporting short or long sleep duration would be less likely to meet PA guidelines. Our second hypothesis was that elderly people living in nursing homes and reporting good sleep quality would be more likely to meet PA guidelines.

## 2. Materials and Methods

### 2.1. Study Participants

In this cross-sectional study, participants were largely independent; they had their own apartments, were taking care of themselves, were free to go out in the city, and were living in a nursing home for the sake of security. In the city of Zagreb, there are 10 nursing homes with approximately 4000 residents in total. In the first stage, we randomly selected five out of ten nursing homes, which had approximately 2000 residents in total. In the second stage, we contacted the principles, head nurses and social workers of each home to help us organize the protocol and collect the data. In order to have 80% power to detect a difference in proportions of 0.15 for meeting PA guidelines between any two groups of sleep duration or sleep quality, where each group comprises one fifth of the sample, in the worst case where the overall proportion is 0.5, and using a two-sided test at the 0.05 level, a total of 870 participants would be needed with usable data. Of 2000 residents, only those who were largely independent and did not need assistance with self-care were considered eligible. Thus, our sample population dropped to 1187. Of these 1187, 153 participants did not want to participate in the study, and 140 participants provided questionnaires with missing data. Hence, our final sample was based on 894 older adults living independently in nursing homes (75% response rate). Data collection in each home was done in groups of 15–20 people. The criteria for participant selection were: age ≥ 65, free of cognitive disability, and physically independent. First, we explained the main purpose of the study and reasons for conducting the study. Second, we briefly explained the risks of the study. All the procedures were anonymous, in accordance with the Declaration of Helsinki, and approved by the Institutional Review Board of the Faculty of Kinesiology (ethics code: 10/7/2018). Before the study began, each participant gave their written informed consent to participation in the study and their approval to use the obtained data for scientific contribution.

### 2.2. PA Assessment

To assess PA over the last seven days, we used the adapted version of the IPAQ–short form, a reliable and valid instrument designed to measure PA in respondents aged ≥65 [21]. The questionnaire elicits information about time spent each day engaging in light, moderate and vigorous intensity PA, and the number of days on which this occurs. For each participant, we calculated the overall time spent doing moderate and vigorous PA.

We categorized participants based on the aforementioned WHO recommendations. Participants who met the recommendations were categorized as “sufficiently” active, while participants who did not meet the recommended levels were categorized as “insufficiently” active.

### 2.3. Sleep Duration

Sleep duration was assessed by asking participants: “On average, how many hours of sleep do you get in a 24h period?” The response was a numerical variable, categorized into five groups for analysis: <6 h (very short sleep), 6–7 h (short sleep), 7–8 h (optimal sleep), 8–9 h (long sleep) and >9 h (very long sleep).

### 2.4. Sleep Quality

To assess sleep quality, we used the Pittsburgh Sleep Quality Index (PSQI) [22]. This is composed of 19 questions which reflect seven major components. All seven components are then summed up to create a scale from 0–21 points. Buysee et al. [22] proposed that the score of <5 denoted “good” sleep quality, while ≥5 denoted “poor” sleep quality. The reliability of the questionnaire in our study was satisfactory (Cronbach’s α = 0.70).

### 2.5. Covariates

Self-rated health was assessed using a one-item question: “How would you rate your health?” Answers were arranged along an ordinal scale as follows: (1) very poor, (2) poor, (3) fair, (4) good, and (5) excellent. We dichotomized the outcome variable into “good” (fair, good and excellent) vs. “poor” (poor and very poor) self-rated health, as done in previous studies [23]. Smoking consumption was categorized as: (1) non-smoker/former smoker vs. (2) present smoker. Alcohol consumption was assessed by one question: “In the past week, did you consume an alcoholic drink?” with “yes” or “no” answers. Psychological distress was assessed by using Kessler’s six-item questionnaire [24]. The scoring protocol was published in detail elsewhere [24]. Kessler et al. [24] showed that responses ≥13 points vs. <13 points respectively distinguished between participants with and without psychological distress. Socioeconomic status was assessed by one-item question: “How would you rate your socioeconomic status?” Responses were arranged along an ordinal scale as follows: (1) below average, (2) average, and (3) above average. The presence or absence of a chronic disease was asked by one-item question: “Have you ever been told by a doctor that you suffer from any kind of chronic disease?” with “yes” and “no” answers. Sex (male or female) was also taken into account.

### 2.6. Data Analysis

Basic descriptive statistics of the study participants are presented as frequencies (n) and percentages (%). Differences between categorical variables were analyzed using Chi-square tests. We used generalized estimating equations to model PA as a binary outcome using logistic regression with clustering at the nursing home level accommodated through an exchangeable working correlation matrix. We also used Pearson’s method for residuals. Because it is plausible that sleep duration and sleep quality are independently associated with PA, we examined the association between sleep duration (7–8 h as referent value) and sufficient PA in model 1, and between sleep quality (poor as referent value) and sufficient PA in model 2. Finally, we entered sleep duration and sleep quality simultaneously into model three, to examine the associations with sufficient PA. All three models were adjusted for sex, self-rated health, smoking consumption, alcohol consumption, psychological distress, socioeconomic status, and chronic disease/s. Significance was set up at α = 0.05 and testing was two-sided (2-sided). All the analyses were performed in Statistical Package for Social Sciences Software, V.22 (IBM).

## 3. Results

At the time this study was conducted, each nursing home had 250–300 users and the univariable analysis revealed no statistical differences in size of nursing homes (*p* = 0.897) between those who did and did not participate. We also tested for potential differences in sleep duration and sleep quality, PA, and covariates between the chosen nursing homes and those not selected using Chi-square tests and found no significant differences between nursing homes (*p* = 0.100–0.783). To analyze for possible selection bias, we used information provided by non-participants regarding their sex, age, body mass index, self-rated health and psychological distress and found no significant differences between participants and non-participants (*p* = 0.247–0.877). The interaction effect of sex was not statistically significant, so we dropped out the sex-stratified analysis (*p* = 0.346).

Basic descriptive statistics of the study participants are presented in Table 1. In general, a higher percentage of short sleepers reported being insufficiently active, while long sleepers were more likely to be sufficiently active, compared to those sleeping 7–8 h. In addition, participants reporting good sleep quality were more likely to be categorized in the sufficiently active category. Those participants who reported having good self-rated health, no alcohol consumption, having low psychological distress and no chronic disease/s were more likely to be categorized as sufficiently active.

The associations of sleep duration and sleep quality with sufficient PA are presented in Table 2. After adjusting for sex, self-rated health, smoking, alcohol consumption, psychological distress, socioeconomic status and chronic disease/s, data were entered separately into model 1 and model 2. Participants reporting short sleep duration (<6 h; OR = 0.45; 95% CI 0.25–0.80) were less likely to report sufficient PA, yet those reporting long sleep duration (>9 h; OR = 2.61; 95% CI 1.35–5.02) (model 1) and good sleep quality (<5 points; OR = 1.59; 95% CI 1.19–2.12) (model 2) were more likely to report sufficient PA. When both variables were entered simultaneously into model three, the same associations remained. Additionally, we wanted to explore whether our covariates had an influence on the associations between sleep duration and sleep quality with PA. After entering only sleep duration and sleep quality separately into the analysis, participants reporting short sleep duration (OR = 0.42; 95% CI 0.24–0.72; *p* < 0.001) were less likely to meet the PA recommendations, yet those reporting long sleep duration (OR = 2.10; 95% CI 1.15– 3.84; *p* = 0.016) and good sleep quality (OR = 1.74; 95% CI 1.33–2.27; *p* < 0.001) were more likely to meet the aforementioned recommendations.

## 4. Discussion

The main purpose of the study was to explore the associations of sleep duration and sleep quality with PA. Our first hypothesis was that both short and long sleep duration would be associated with not meeting the PA recommendations for elderly people aged ≥65. However, our results only partially confirmed the hypothesis, as participants who reported sleeping <6 h were less likely to meet the PA recommendations, yet those reported sleeping >9 h were more likely to meet the PA recommendations. Previous studies have reported that short sleep duration is associated with lower PA levels in elderly populations [8]. However, one other study showed no significant association between sleep duration and PA in individuals who reported fewer depressive symptoms [25]. In general, previous studies have shown that short sleep duration is associated with a decrease in maximal oxygen uptake [26], an increase in exercise-related injuries [27] and daytime tiredness and fatigue [28], leading to reduced regular participation in PA.

Contrary to our first hypothesis, our results showed that elderly individuals who reported sleeping >9 h were more likely to be sufficiently active. A study by Garfield et al. [25] showed similar results, although an association was observed only in elderly individuals who reported having depressive symptoms. One study conducted among Chinese adolescents showed that long sleepers were more likely to participate in ≥60 minutes of PA on ≥5 days/week. A systematic review by Kredlow et al. [29] showed that the engagement in PA, although small, had a significant beneficial effect on total sleep time. Thus, PA may promote longer sleep time, especially in elderly individuals.

Finally, our second hypothesis stated that good sleep quality would be associated with sufficient PA in the elderly. The results of our study confirmed this hypothesis and are in line with previous studies conducted among the same population [13,30,31]. Specifically, two cross-sectional studies [30,31] have shown that good subjective sleep quality was positively associated with PA. Moreover, a longitudinal study by Holfeld and Ruthig [13] showed that better initial sleep quality predicted higher levels of later PA, yet initial PA did not predict later sleep quality. However, the physiological basis of such associations is not yet fully understood. Physiological changes during PA, such as increased body temperature, improved heart rate, and decreased psychological distress may all improve sleep quality [32], yet some studies suggest that the association is bidirectional [14].

The reason we used both sleep duration and sleep quality as predictors of PA was that both variables have been consistently associated with each other and it has been proposed that both sleep duration and sleep quality should be taken into account for public health [33]. Also, both variables have been consistently associated with similar negative health-related outcomes [9,11,12,13].

Our study has several strengths. First, we randomly selected five nursing homes and conducted a study among a relatively high number of individuals (n = 894). Second, we used previously validated questionnaires to assess PA, sleep duration, and sleep quality. Third, all three models were adjusted for sex, self-rated health, smoking, alcohol consumption, psychological distress, socioeconomic status and chronic disease/s.

However, our study has several limitations. Due to a cross-sectional design, the associations between sleep duration and sleep quality with PA must be interpreted with caution. However, previous research has shown that the association between sleep and PA is bidirectional [14]. Although we used validated questionnaires, our second limitation was the use of self-reported measures. It is possible that individuals over- or under-reported their PA levels and sleeping variables, which could have led to considerable measurement error, recall bias, and social desirability effect [34]. Third, we were lacking in information about physiological parameters and also daylight exposure, which has a beneficial effect on well-being and psychological functioning [35]. Fourth, we based our sample only in the elderly individuals situated in nursing homes in the city of Zagreb. However, free-living individuals might have had different levels of PA and sleep, leading to different associations. Finally, we put both sleep duration and sleep quality simultaneously in the analysis and found significant associations with PA. However, it is possible that the only significant predictor of PA is sleep duration and the association with the sleep quality measure is only being driven by that. Future studies should use objective methods (accelerometry and polysomnography) and longitudinal methodology in order to better capture and understand the causality between PA and sleep.

## 5. Conclusions

Our study shows that elderly people reporting short sleep duration are less likely to meet the PA recommendations for their age group, yet those reporting long sleep duration and good sleep quality are more likely to meet the same recommendations. These results present some new evidence of the independent associations between sleep duration and sleep quality with PA in this population. Our findings suggest that long sleepers and those report good sleep quality are more likely to meet PA recommendations.

## Figures and Tables

**Table 1:** Basic descriptive statistics of the study participants (*N* = 894).

Study Variables	Total (*N* = 894)	‘Sufficiently’ Active (*N* = 389)	‘Insufficiently’ Active (*N* = 505)	*p*-Value *
	*N* (%)	*N* (%)	*N* (%)	
Sleep duration				
<6	76 (8.5)	20 (5.1)	56 (11.1)	
6–7	150 (16.8)	55 (14.1)	95 (18.8)	
7–8	486 (54.4)	223 (57.3)	263 (52.1)	
8–9	132 (14.8)	59 (15.2)	73 (14.5)	
>9	50 (5.6)	32 (8.2)	18 (3.6)	<0.001
Sleep quality				
Poor	487 (54.5)	184 (47.3)	303 (60.0)	
Good	407 (45.5)	205 (52.7)	202 (40.0)	<0.001
Sex				
Men	393 (44.0)	229 (58.9)	164 (32.5)	
Women	501 (56.0)	160 (41.1)	341 (67.5)	<0.001
Self-rated health				
Poor	132 14.8)	35 (9.0)	97 (19.2)	
Good	762 (85.2)	354 (91.0)	408 (80.8)	<0.001
Smoking consumption				
Yes	281 (31.4)	113 (29.0)	168 (33.3)	
No	613 (68.6)	276 (71.0)	337 (66.7)	0.191
Alcohol consumption				
Yes	227 (25.4)	81 (20.8)	146 (28.9)	
No	667 (74.6)	308 (79.2)	359 (71.1)	0.007
Psychological distress				
High	124 (13.9)	29 (7.5)	95 (18.8)	
Low	770 (86.1)	360 (92.5)	410 (81.2)	<0.001
Socioeconomic status				
Low	33 (3.7)	15 (3.9)	18 (3.6)	
Middle/high	861 (96.3)	374 (96.1)	487 (96.4)	0.859
Chronic Disease/s				
Yes	115 (12.9)	39 (10.0)	76 (15.0)	
No	779 (87.1)	350 (90.0)	429 (85.0)	0.027

* Chi-square test.

**Table 2:** The associations of sleep duration and sleep quality with sufficient physical activity (PA) in the study participants (*N* = 894).

Study Variables	Model 1 (*N* = 894)	Model 2 (*N* = 894)	Model 3 (*N* = 894)
	OR (95% CI; *p*-Value)	OR (95% CI; *p*-Value)	OR (95% CI; *p*-Value)
Sleep duration			
<6	0.50 (0.27 to 0.83; 0.007)		0.52 (0.30 to 0.91; 0.025)
6–7	0.79 (0.51 to 1.18; 0.310)		0.93 (0.61 to 1.40; 0.671)
7–8	Ref.		Ref.
8–9	1.25 (0.83 to 1.90; 0.335)		1.22 (0.79 to 1.85; 0.320)
>9	2.64 (1.40 to 5.06; <0.001)		2.58 (1.35 to 4.92; <0.001)
Sleep quality			
Poor		Ref.	Ref.
Good		1.61 (1.23 to 2.11; <0.001)	1.47 (1.10 to 1.97; 0.021)
Sex			
Men	Ref.	Ref.	Ref.
Women	0.31 (0.22 to 0.43; <0.001)	0.34 (0.25 to 0.43; <0.001)	0.33 (0.27 to 0.45; <0.001)
Self-rated health			
Poor	Ref.	Ref.	Ref.
Good	1.80 (1.14 to 2.88; 0.017)	1.64 (1.05 to 2.58; 0.043)	1.68 (1.07 to 2.60; 0.034)
Smoking consumption			
Yes	Ref.	Ref.	Ref.
No	1.06 (0.76 to 1.46; 0.649)	1.05 (0.77 to 1.44; 0.825)	1.06 (0.77 to 1.46; 0.705)
Alcohol consumption			
Yes	Ref.	Ref.	Ref.
No	1.39 (1.00 to 1.94; 0.048)	1.45 (1.05 to 2.00; 0.024)	1.43 (1.05 to 2.07; 0.031)
Psychological distress			
High	Ref.	Ref.	Ref.
Low	2.15 (1.33 to 3.44; <0.001)	2.00 (1.23 to 3.22; <.001)	1.94 (1.20 to 3.15; <0.001)
Socioeconomic status			
Low	Ref.	Ref.	Ref.
Middle/high	0.71 (0.32 to 1.54; 0.421)	0.74 (0.35 to 1.60; 0.451)	0.73 (0.34 to 1.59; 0.436)
Chronic disease/s			
Yes	Ref.	Ref.	Ref.
No	1.10 (0.71 to 1.72; 0.628)	1.11 (0.71 to 1.75; 0.676)	1.10 (0.70 to 1.74; 0.664)

Model 1: Examine the association between sleep duration and sufficient PA adjusted for sex, self-rated health, smoking consumption, alcohol consumption, psychological distress, socioeconomic status and chronic disease/s. Model 2: Examine the association between sleep quality and sufficient PA adjusted for the same variables. Model 3: Examine the associations of sleep duration and sleep quality with sufficient PA adjusted for the same variables.

## Abbreviations

95% CI	95 percent confidence interval
IPAQ	International Physical Activity Questionnaire
OR	odds ratio
PA	physical activity
PSQI	Pittsburgh Sleep Quality Index
WHO	World Health Organization
